# Neurocognitive Predictors of Treatment Outcomes in Cognitive Processing Therapy for Post-traumatic Stress Disorder: Study Protocol

**DOI:** 10.3389/fpsyg.2021.625669

**Published:** 2021-01-26

**Authors:** David P. Cenkner, Anu Asnaani, Christina DiChiara, Gerlinde C. Harb, Kevin G. Lynch, Jennifer Greene, J. Cobb Scott

**Affiliations:** ^1^VISN4 Mental Illness Research, Education, and Clinical Center, Corporal Michael J. Crescenz VA Medical Center, Philadelphia, PA, United States; ^2^Department of Psychology, University of Utah, Salt Lake City, UT, United States; ^3^Department of Psychiatry, Perelman School of Medicine, University of Pennsylvania, Philadelphia, PA, United States

**Keywords:** PTSD—post-traumatic stress disorder, memory, psychotherapy research, psychotherapy mechanisms, cognitive functioning

## Abstract

**Background:**

Post-traumatic stress disorder (PTSD) is a prevalent, debilitating, and costly psychiatric disorder. Evidenced-based psychotherapies, including Cognitive Processing Therapy (CPT), are effective in treating PTSD, although a fair proportion of individuals show limited benefit from such treatments. CPT requires cognitive demands such as encoding, recalling, and implementing new information, resulting in behavioral change that may improve PTSD symptoms. Individuals with PTSD show worse cognitive functioning than those without PTSD, particularly in acquisition of verbal memory. Therefore, memory dysfunction may limit treatment gains in CPT in some individuals with PTSD.

**Methods and Analysis:**

Here, we present a protocol describing the Cognition and PsychoTherapy in PTSD (CPTPTSD) study, a prospective, observational study examining how cognitive functioning affects treatment response in CPT for PTSD (NCT# 03641924). The study aims to recruit 105 outpatient veterans with PTSD between the ages of 18 and 70 years. Prior to beginning 12 sessions of CPT, Veteran participants will have standardized assessments of mood and functioning and complete a comprehensive neurocognitive battery assessing episodic learning, attention and speed of processing, language ability, executive control, and emotional functioning. This study aims to fill gaps in the current literature by: (1) examining the specificity of memory effects on treatment response; (2) exploring how baseline cognitive functioning impacts functional outcomes; and (3) examining potential mechanisms, such as memory for treatment content, that might explain the effects of baseline memory functioning on PTSD symptom trajectory.

**Discussion:**

If successful, this research could identify clinically relevant neurocognitive mechanisms that may impact PTSD psychotherapy and guide the development of individualized treatments for PTSD.

## Introduction

Individuals who experience combat, sexual trauma, and other traumatic events are at higher risk for developing post-traumatic stress disorder (PTSD). PTSD is the most common mental health diagnosis received by returning Iraq and Afghanistan (OEF/OIF/OND) veterans at Veteran’s Administration (VA) facilities (Seal et al., 2007), with lifetime prevalence estimates ranging from 8 to 20% in OEF/OIF veterans (Hoge et al., 2006) and 15 to 30% in Vietnam veterans (Dohrenwend et al., 2006). Within VA, roughly 20% of Veterans who endorse military sexual trauma (MST) also have a diagnosis of PTSD (Gilmore et al., 2016). PTSD is frequently chronic (Kessler et al., 1995) and is often associated with impaired functioning (Byers et al., 2014). Costs for PTSD (e.g., work impairment) are substantial. For example, between 2004 and 2012, total expenditures for PTSD rose from 29.6 to 294.1 million (Institute of Medicine, 2014). Taken together, the above considerations illustrate the urgent need to develop effective treatments for PTSD.

Cognitive Processing Therapy (CPT) is a trauma-focused psychotherapy strongly recommended for the treatment of PTSD (Resick et al., 2017). CPT is among the frontline trauma-focused psychotherapies recommended by the existing VA/DoD Clinical Practice Guidelines for the management of PTSD (Department of Veterans Affairs and Department of Defense, 2017). CPT is a protocoled cognitive-behavioral therapy which emphasizes cognitive therapy techniques to help challenge erroneous beliefs developed or strengthened by traumatic events that are maintaining PTSD symptoms and preventing natural recovery from trauma. The goal of CPT is to help patients recover from PTSD symptoms by reconciling unhelpful beliefs and unprocessed emotions from trauma through both acceptance and the development of more balanced, adaptive beliefs. Unfortunately, a fair proportion of individuals show limited benefit from a standard course of psychotherapy, including CPT, for PTSD (Larsen et al., 2019). A recent meta-analysis of PTSD psychotherapies in veterans showed that a majority of patients receiving treatments show clinically significant improvements, but most still meet diagnostic criteria for PTSD, and non-response rates are not insignificant (Steenkamp et al., 2015). To develop and refine interventions for individuals with PTSD, there is thus a need to identify factors amenable to intervention that may further enhance psychotherapy outcomes for this disorder.

One factor that may impact psychotherapy in PTSD is cognitive functioning. Psychotherapy requires patients to encode and consolidate new information, organize this information into behavioral plans, and remember and execute these plans. Thus, the capability to retrieve specific details from one’s past and encode/retrieve treatment-relevant skills are highly relevant for therapeutic change across most types of psychotherapy (Liggan and Kay, 1999). Critically, numerous studies have shown that individuals with PTSD show poorer cognitive functioning than individuals without PTSD (Schuitevoerder et al., 2013; Scott et al., 2015), including deficits in episodic memory (Brewin et al., 2007), speed of information processing (Wrocklage et al., 2016), attention (Vasterling et al., 2002), and executive functioning (Aupperle et al., 2012), with the largest effects observed in immediate verbal memory (i.e., learning) (Scott et al., 2015). Treatment-seeking individuals with PTSD appear to have greater cognitive deficits compared to community-based PTSD samples (Scott et al., 2015), highlighting the potential relevance of these deficits for clinical practice. Moreover, cognitive deficits are uniquely associated with negative functional outcomes in PTSD, including occupational performance, social functioning, and quality of life (Geuze et al., 2009; Wrocklage et al., 2016). However, despite its potential relevance, cognitive functioning is only beginning to be explored as an influential factor in psychotherapy outcomes for PTSD.

Accumulating research reports remarkably consistent effects of verbal memory deficits on psychotherapy outcomes in PTSD. In a small study of civilians in trauma-focused CBT, Wild and Gur (2008) reported that non-responders had significantly worse verbal learning (i.e., immediate memory) at baseline, and that improvement in PTSD symptoms was positively associated with verbal learning performance, even after accounting for pre-treatment PTSD severity and depression. In a larger study of 140 individuals with PTSD, Nijdam et al. (2015) showed that immediate verbal memory was positively associated with PTSD remission and with degree of change in PTSD severity over 16 weeks of treatment. Haaland et al. (2016) replicated these findings in a small sample of women veterans, showing that verbal memory at baseline was a predictor of psychotherapy response during group therapy with elements of both CPT and Prolonged Exposure (PE), another front-line evidence-based psychotherapy (EBP) for PTSD. Furthermore, a study of sleep and nightmare treatment in 94 veterans with PTSD showed that individuals with lower verbal learning performance at baseline were less likely to respond to treatment (Scott et al., 2017). Together, the consistency and robustness of these findings have prompted recommendations that verbal memory could serve as a useful and readily obtainable predictor of psychotherapy treatment response in PTSD (Etkin, 2015). However, limited research has examined the specificity of memory dysfunction in predicting treatment outcomes compared to overall cognitive functioning.

Furthermore, despite accumulating research in this area, no studies to date have examined the pathways by which cognitive functioning may affect treatment outcomes. For example, one pathway by which cognitive dysfunction may interfere with psychotherapy is by directly limiting treatment understanding, learning, or implementation, or interfering with a patient’s ability to re-contextualize memories. Evidence from research in substance use disorders and depression indicates that worse executive functioning, memory, and overall cognitive abilities are associated with poorer recall of treatment information (Teichner et al., 2002; Harvey et al., 2016), reduced quality of coping skills acquired (Alexopoulos et al., 2003), and fewer treatment objectives achieved (Teichner et al., 2001). However, no studies have been conducted in stress-related disorders to determine which aspects of treatment are most affected by cognitive deficits. To address this limitation, the proposed project aims to test specific models of pathways between memory dysfunction and treatment outcomes by integrating novel insights from both cognitive neuropsychology and psychotherapy research.

In sum, accumulating evidence indicates that memory dysfunction is prominent in PTSD and adversely affects psychotherapy outcomes. Identifying mechanisms underlying the relationship between memory dysfunction and poor treatment outcomes is crucial to provide empirical guidance regarding appropriate rehabilitation targets to reduce the impact of cognitive dysfunction on therapy outcomes. The specific rehabilitation approaches that are most appropriate for improving therapy outcomes in PTSD will depend on the specific deficits observed, their relationship with therapeutic and functional outcomes, and critical moderators or mediators of those relationships. Given convergent findings of associations between cognitive functioning and PTSD and its influence on psychotherapy outcomes, the current protocol was developed to provide a rigorous examination of such relationships using hypothesis-driven models. Notably, no prior studies have examined effects of cognitive functioning on psychotherapy outcomes as a primary aim or explored potential mechanisms underlying these relationships. The current protocol therefore aims to address several knowledge gaps in the literature.

First, we aim to examine the specificity of memory effects on PTSD symptom response and functional outcomes after CPT. Our primary hypothesis is that verbal immediate memory (i.e., learning), but not overall cognitive performance, will interact with time to predict PTSD symptom changes at post-treatment, such that those with lower verbal learning performance at baseline will have less reduction in PTSD symptoms than those with higher verbal learning performance. Second, we aim to determine whether specific treatment-related variables (e.g., memory for treatment content, treatment adherence) mediate the relationship between memory functioning and CPT response, helping to explain *how* episodic memory functioning might lead to poor treatment outcomes. Our primary hypothesis for this aim is that treatment recall at the treatment midpoint will mediate the association between verbal immediate memory at baseline and treatment outcomes. Finally, in an exploratory fashion, we also aim to examine whether alternative models of dysfunctional memory in PTSD, including impairment in autobiographical memory (Barry et al., 2018), emotional memory consolidation (Durand et al., 2019), or prospective memory (Scott et al., 2016), might better explain the relationship between memory deficits and psychotherapy outcomes. For this aim, our primary hypothesis is that effect size comparisons will reveal that verbal learning functioning at baseline exhibits larger effects than prospective memory, emotional memory consolidation, or autobiographical memory in predicting psychotherapy treatment outcomes. If successful, data from this study could lead to a shift in clinical psychotherapy practice for veterans with PTSD and cognitive dysfunction by identifying salient, modifiable factors that may impact treatment outcomes.

## Methods and Analysis

### Design

The Cognition and PsychoTherapy in PTSD: Mechanisms and Functional Outcomes (CPTPTSD; NCT# 03641924) study is an observational, prospective study aiming to recruit 140 (*n* = 105 after attrition; see Missing Data below) outpatient veteran participants aged between 18 and 70 who meet criteria for PTSD. Veteran outpatients are being recruited from the Corporal Michael J. Crescenz Veterans Affairs Medical Center (CMCVAMC) in Philadelphia, Pennsylvania.

### Participants

Participants include veterans who are being referred for EBPs for PTSD (e.g., CPT, PE) within the outpatient mental health clinic at CMCVAMC. Participants are referred to the study following evaluation from a behavioral health technician, in lieu of transfer to standard outpatient psychotherapy appointment for an EBP for PTSD, or by a pre-existing outpatient mental health provider. Inclusion criteria includes meeting DSM-5 criteria for PTSD via the Clinician Administered PTSD Scale (CAPS-5) and being between the ages of 18 and 70. Exclusion criteria includes moderate or severe substance use disorder (SUD) not in remission for >1 month; daily benzodiazepine use; severe psychiatric illness (e.g., schizophrenia); significant current suicidal or homicidal intent, including a specific plan; dementia, neurological disorder, or severe TBI (i.e., loss of consciousness >24 h); inability to speak or read English; prior completion of CPT; and psychiatric hospitalization within 30 days of study entry. To enhance generalizability among our sample of veterans with PTSD, participants are allowed to concurrently take medications for PTSD and other mental (e.g., depression) or physical health conditions or attend concurrent talk or supportive therapy.

### Procedure

The first recruitment of veteran participants started in July 2019 with recruitment expected to be completed in March 2023. Participants undergo study screening to determine initial eligibility. Major assessments are conducted with participants at study entry (baseline), session 4, session 6, session 8, and at an endpoint assessment at post-treatment. For a full breakdown of all assessments administered at these time points (see Table 1). Baseline procedures (before starting CPT) include diagnostic interviews, questionnaires assessing mood and functioning, a neurocognitive assessment, and exploratory memory measures. After baseline, participants answer questionnaires about treatment expectancy and begin a standard course of CPT (12 sessions) following standardized protocols (Resick et al., 2017) with our study therapists, who are all certified CPT providers within the VA healthcare system. Study therapists are provided regular supervision focused on CPT protocol adherence and fidelity during the course of the study. Therapists are blind to scores on cognitive measures, and all CPT sessions are recorded for fidelity monitoring.

**TABLE 1:** Schedule of measure administration.

	Baseline	Post-CPT session 1	Post-CPT session 4	Post-CPT session 6	Post-CPT session 8	End of CPT
**Interview measures**						
MINI	•					
CAPS-5	•					•
LEC-5	•					
Social/Medical history	•					
CES	•					
OSU TBI-ID	•					
**Self-report measures**						
PCL-5	•		•	•	•	•
SAS-SR	•		•	•	•	•
RAND-12 QOL	•		•	•	•	•
WHO-HPQ	•		•	•	•	•
PHQ-9	•	•	•	•	•	•
**Cognitive assessments**						
Neurocognitive Tests	•					•
**Treatment measures**						
TARS			•	•	•	•
PRT			•	•	•	•
ETO		•		•		
CEQ		•		•		
SES		•		•		•

**MINI, The Mini-International Neuropsychiatric Interview; CAPS-5, The Clinician Administered PTSD Scale-5; CES, Combat Experiences Scale; OSU TBI-ID, Ohio State University Traumatic Brain Injury Identification; PCL-5, The PTSD Checklist for DSM-5; SAS-SR, The Social Adjustment Scale-Self Report; RAND-12 QOL, Veterans RAND 12 Item Health Survey; WHO-HPQ, WHO Work Performance Questionnaire; PHQ-9, The Patient Health Questionnaire-9; TARS, Treatment Adherence Rating Scale; PRT, Patient Recall Task; ETO, Expectancy of Therapeutic Outcome; CEQ, Credibility/Expectancy Questionnaire; SES, Self-Efficacy Scale. Bold dots mean that the measure is administered at that timepoint.**

Following each CPT visit, participants complete a brief questionnaire about adherence and application of treatment (see below), as well as the Patient Health Questionnaire (PHQ-9; Kroenke et al., 2001) to assess depression symptoms and suicide risk. At CPT sessions 4, 6, 8, and 12, participants complete measures of memory for treatment and brief re-assessments of PTSD, mood, and functioning.

Note that with the outbreak of the novel Coronavirus-19 (COVID-19) in March of 2020, all study procedures have moved to a virtual platform, and verbal consent is being conducted, as approved by the CMC VAMC IRB.

### Interview Assessments

During the baseline visit, participants are interviewed by a trained and experienced masters-level clinician using the: (1) Clinician Administered PTSD Scale-5 (Weathers et al., 2013a), the gold standard interview for diagnosing PTSD; (2) Mini International Neuropsychiatric Interview (MINI) for DSM-5 (Sheehan et al., 2015), a semi-structured interview that yields DSM-5 Axis I diagnoses for major psychiatric disorders, used for both sample characterization and exclusionary criteria; (3) Life Events Checklist-5 (Weathers et al., 2013b), to properly identify an index trauma; (4) structured interview for sociodemographic and medical history to obtain relevant demographic information and medical history; and (5) Ohio State University TBI Identification (Corrigan and Bogner, 2007), a brief structured interview to assess for previous signs of traumatic brain injury, for characterization of the sample with regard to head injuries. The CAPS is also readministered to each participant at the final visit.

### Self-Report Measures

Two assessments are administered to capture primary outcomes: (1) PTSD Checklist for DSM-5 (PCL-5; Weathers et al., 2013c) to assess PTSD symptoms; and (2) the Social Adjustment Scale-Self Report (SAS-SR; Weissman, 1999), a 54-item measure assessing the six domains of work, leisure, extended family, romantic relationships, parental, and family unit functioning. Two assessments are administered to capture secondary outcomes: (1) the Veterans RAND 12-Item Health Survey (RAND-12; Kazis et al., 2006) to measure health-related quality of life; and (2) the WHO and Work Performance Questionnaire (HPQ; Kessler et al., 2004) to measure performance at work and hours missed from work over the previous month.

Two instruments are administered to capture covariate and predictor outcome variables. First, the PHQ-9, as mentioned previously, assesses depressive symptoms. Second, the Combat Experiences Scales (CES; Vogt et al., 2013) is used to characterize participant combat exposure.

### Neurocognitive Assessments

The neurocognitive battery assesses participants’ functioning across a broad range of neurocognitive domains using both traditional paper and pencil testing and computerized measures from the Penn Computerized Neurocognitive Battery (PennCNB; Gur et al., 2001, 2010), a widely administered battery with substantial evidence of reliability and validity (Gur et al., 2012; Moore et al., 2015; Swagerman et al., 2016). Specifically, episodic learning and memory are measured using the California Verbal Learning Test-2 (CVLT-II; Delis et al., 2000), WMS-IV Logical Memory Test (Wechsler, 2009), and the Penn Face Memory Test (Thomas et al., 2013). Attention and speed of processing is measured using the Penn Continuous Performance Test (Kurtz et al., 2001), Trailmaking Test Part A (Bowie and Harvey, 2006), and the Penn Digit Symbol Test (Bachman et al., 2010). Language ability is measured using the Penn Verbal Reasoning Test (Bilker et al., 2014). Executive control is measured using the Trailmaking Test Part B (Bowie and Harvey, 2006), Stroop Color-Word Interference Test (MacLeod, 1991), Penn Conditional Exclusion Test (Kurtz et al., 2004), and the Letter N-Back Test (Ragland et al., 2002). Emotional Functioning is measured using the Penn Emotion Recognition Test (Gur et al., 2001) and the Penn Emotion Discrimination Test (Gur et al., 2001). Performance Validity is measured using the Medical Symptom Validity Test (MSVT; Green, 2004). Finally, IQ is estimated using the Wechsler Test of Adult Reading (WTAR; Wechsler, 2001). The entire neurocognitive battery is administered by research coordinators who undergo extensive training, and measures are administered in a secluded and quiet room with adequate space for participant and coordinator. These measures are administered during the baseline visit prior to the onset of CPT and after the final session CPT visit.

### Measures of Treatment Processes

Presently, there are no ideal measures with extensive evidence of reliability and validity to assess treatment adherence, memory for treatment content, and treatment expectancy. Thus, measures with strong theoretical foundations and initial evidence of validity were chosen for administration. Treatment adherence is measured weekly by the Therapist- and Patient-Report versions of the Treatment Adherence Rating Scale (TARS; Dong et al., 2016). The TARS assesses (1) if the treatment was comprehended and accepted by the patient as intended, and assesses patient acceptance and agreement with the session content. The TARS is administered weekly throughout treatment. Patient recall of treatment content is measured using the standardized Patient Recall Task (PRT; Lee and Harvey, 2015). The PRT is a free recall task that has patients recall as many treatment points as they can remember in 10 min. The PRT is administered during the midpoint and end of study sessions.

Treatment expectancy is captured through three measures, including: (1) Expectancy of Therapeutic Outcome (ETO; Strauss et al., 2018), a 5-item Likert scale aimed to evaluate treatment credibility after the therapist provides the treatment rationale; (2) Credibility/Expectancy Questionnaire (CEQ; Devilly and Borkovec, 2000), a 6-item Likert scale measuring treatment expectancies for success; and (3) Self-Efficacy Scale (SES; Brown et al., 2014), a one item questionnaire that asks the participant to rate his or her capability for successfully participating in treatment.

### Additional Memory Measures

Participants also receive three additional measures of memory processes shown to be dysfunctional in PTSD, including the Autobiographical Memory Test (AMT; Mark and Broadbent, 1986), the Emotional Verbal Learning Test (EVLT; Strauss and Allen, 2013), and a modified version of the Memory for Intentions Test (MIST; Raskin et al., 2010). The AMT includes 12 positive and negative words relevant to depression and anxiety, each presented at once, and asks the participant to write a specific memory he or she associates with the provided word. Participants are given up to 2 min for each word. The EVLT asks participants to recall words presented from four emotion categories (happiness, sadness, anger, and anxiety) over five trials and delayed free recall. The modified MIST is a performance-based test of prospective memory (i.e., remembering to remember) over 15 min with time-based cues balanced by mode of response (i.e., physical vs. verbal) and with variable delay periods (e.g., 2, 10 min).

### Data Analysis

Statistical models used are linear or generalized linear mixed effects regression models, and we will use standard residual-based diagnostic approaches to check that model assumptions are satisfied (e.g., regarding outliers, observations with high influence or leverage). We will examine demographics as covariates in analyses to account for possible confounding of memory effects by demographics (e.g., age).

We will use mixed effects models to examine the effects of verbal learning on overall PTSD symptom response, and on overall functioning. Verbal learning will be represented by a mean *z*-score of CVLT-2 Trials 1–5 and WMS Logical Memory I. The comparison will be based on repeated responses obtained at baseline, mid-point, and end of treatment. The model for each outcome will include a random intercept to accommodate between-subject heterogeneity (Fitzmaurice et al., 2012). We anticipate that the two primary responses can be modeled as continuous measures, and that a linear model will fit the data well. If necessary, we will use generalized linear mixed effects models to accommodate skewness or departures from normality and linearity. The primary explanatory variables in the models will be baseline verbal learning score and time, and their interaction. Time will be coded as a categorical factor representing baseline, mid-treatment, and end-of-treatment. The primary analysis will be intent-to-treat, in which participant data is analyzed regardless of dropout or adherence status, as mixed effects models accommodate partial data provided by non-completers. The main effects will test whether veterans with lower verbal learning performance will display less reduction in PTSD symptoms (PCL-5) across treatment, and less improvement in overall functioning (measured with the SAS-SR), than those with better verbal learning performance. The interaction effects will test for variations in these effects across the treatment phase. A further question of interest relates to how prediction based on memory-specific aspects is related to prediction based on overall cognitive functioning. To address this question, we will generate a measure of overall cognitive performance from the other elements in the neurocognitive test battery. To facilitate data reduction, and since the PennCNB has been shown to have a strong general factor in prior research (Moore et al., 2015; Swagerman et al., 2016), we will perform a principal component analysis (PCA) for all neurocognitive measures except for the memory measures. We will examine the strength of the association between this principal component and the memory measures to determine whether the first principal component appears to be a reasonable measure of overall cognitive functioning.

Second, to examine whether mid-treatment levels of treatment engagement (TARS) and memory for treatment (PRT) mediate the effects of baseline verbal learning on end of treatment PTSD symptoms and overall functioning, we will use a multiple mediator model following MacKinnon (2008, Equations 5.2–5.4) to assess effects of verbal learning and the two mediators simultaneously. This approach provides the most direct test of our explicit hypothesis of mediation but does not make use of the full set of longitudinal data available on the mediators and outcomes. Thus, we will supplement it with a set of longitudinal models, which provide more information on relationships between the time-course of repeated-measures variables. We do not measure verbal learning during treatment, as it is not expected to substantially change over the treatment period (Haaland et al., 2016). Thus, we will fit simplified versions of latent growth curve models, fitting simultaneous growth curves to mediators and responses, and using verbal learning as a non-time-varying baseline covariate. These models can accommodate different numbers of time points, and different timepoints, across the mediators and responses. Based on the number of time points (4–6, depending on mediator and outcome), we expect that linear growth curves will be sufficient to describe the trajectories. Our primary model will consider the PTSD outcome and the treatment recall mediator. Our model will allow verbal learning to have direct effects on the random intercepts and slopes of the recall and PTSD processes, for the PTSD random intercept to have a direct effect on the recall slope, and for the recall intercept and slope to have direct effects on PTSD slope. The mediation effect is then the estimated by the product of the estimated direct effect of verbal learning on the recall slope, and the direct effect of the recall slope on the PTSD slope.

Third, to determine whether aspects of memory are better predictors of treatment and functional outcomes than verbal memory in exploratory analyses, similar mixed effects models as the primary analyses will be conducted to examine the effects of baseline autobiographical memory, emotional memory, and prospective memory on overall PTSD symptom response.

Finally, a small set of additional covariates potentially associated with PTSD psychotherapy outcomes, including TBI status and depression severity, will be included in reruns of our primary analyses. Model selection approaches based on BIC model fit comparisons to examine the separate and joint variance explained by primary and additional covariates will be used. We will also examine models with interactions between verbal memory and these additional covariates to assess whether they act as moderators of memory effects. Although exploratory in nature, these covariates will be included as baseline variables in the latent growth mediation models.

### Missing Data

Based on our team’s prior studies, we anticipate a loss of up to 25% of veterans who enroll but do not start treatment (note that this study does not involve randomization). Thus, our planned enrollment is *n* = 140, although the sample size of those who initiate CPT is expected to be *n* = 105.

Sporadic missing items from a scale may yield a small amount of missing data. We will accommodate this in analyses by mean imputation of a total scale score, based on available items of all participants at that time point. Mixed-effects models and longitudinal mediation analyses make use of all data provided by participants. The analysis on the combined set of complete and incomplete data will be valid if the missing-at-random (MAR) assumption is met; in practical terms, this means that a future missed visit can be well-predicted on the basis of available data. We will perform sensitivity analyses using selection models (Fitzmaurice et al., 2012) to assess effects of plausible models for non-MAR dropout on primary hypotheses. We will use mixed-effects logistic regression models to predict drop at each timepoint, using covariates available at that point. These models will provide estimated probabilities of attendance at the timepoints, and we will use (standardized) versions of these probabilities as inverse-weights in the analyses for the Aims. For non-longitudinal mediation analyses, we will use a multiple imputation approach to generate ten data sets with missing responses imputed for all participants, estimate the models for the secondary hypotheses on each dataset, and combine the results to get final estimates that accommodate the variability due to imputation.

### Power Analysis/Sample Size

We regard our hypothesis on PTSD symptoms as separate from hypotheses on functioning, so we use a 5% alpha-level for both outcomes. Prior data (Nijdam et al., 2015; Haaland et al., 2016; Scott et al., 2017) suggest a time by verbal learning interaction during treatment, where those with higher baseline verbal learning show greater reduction in PTSD related outcomes than people with lower baseline verbal learning and retain much of the improvement. In the proposed study, we anticipate that the difference will increase steadily through the full treatment phase. We base power estimates on the data of Scott et al. (2017) and our pilot data (Scott et al., 2015). From both of those data, using *z*-scored versions of baseline verbal learning and the response, a one unit increase in verbal learning corresponded to an additional 0.15 unit decrease in the estimated change in PTSD outcomes. Based on 500 simulated datasets, we have 80% power for that observed effect for the PTSD outcome, and 81% power for slightly larger effects (additional decreases of 0.18 per time period) for functioning measures. For mediation hypotheses of Aim 2, we use the methods of Fritz and MacKinnon (2007). Using a 5% alpha-level, our attrition reduced sample of 105 yields 80% power for scenarios with medium or larger effects for the effect of verbal learning on a mediator, and the effect of the mediator on the outcome.

## Discussion

This protocol paper describes an observational prospective study to examine the relationship between neurocognitive mechanisms and CPT outcomes in a sample of veterans with PTSD. Prior research has identified cognitive deficits in individuals with PTSD, with the largest effects in immediate verbal memory (Scott et al., 2015). Moreover, secondary analyses of PTSD psychotherapy trials have consistently shown that verbal memory impacts treatment outcomes across settings and samples. To our knowledge, the CPTPTSD study will be the first to examine whether particular treatment-related variables may mediate the impact of memory on PTSD outcomes. Results will identify specific mechanisms underlying the relationship between cognitive functioning and psychotherapy outcomes in PTSD, which will inform future rehabilitation interventions that specifically target these cognitive deficits and their downstream effects. Identifying such targets are essential for developing effective clinical interventions to mitigate the effects of memory and other cognitive deficits on psychotherapy treatment response.

### Limitations

This study has limitations. First, the protocol is not a randomized controlled trial (RCT), and therefore we will not be able to definitively know if symptom changes during CPT that are associated with neurocognitive functioning would have happened absent any intervention. However, conducting an RCT was not necessary to examine most relationships posed in our hypotheses. Second, we have faced numerous challenges with the outbreak of COVID-19, yet we have innovated and effectively transitioned much of our protocol to a virtual platform. Lastly, our study population is comprised solely of veterans, and results may not generalize to the civilian population. However, there is evidence that veteran and civilian PTSD rates are similar after experiencing similar traumas (Patel et al., 2016), and there are minimal differences in cognitive functioning in veteran vs. civilian samples with PTSD (Scott et al., 2015).

## Ethics and Dissemination

This project was approved and carried out with feedback from the Corporal Michael J. Crescenz Veterans Affairs Institutional Review Board. All subjects will provide written or verbal consent prior to enrollment into the study.

Findings from this research will be disseminated in peer-reviewed journals and scientific meetings. We will also communicate results and implications to critical stakeholders in the veteran and PTSD research communities. Results will also inform the long-term goal of this program of research, which is to understand the role of cognitive dysfunction in psychotherapy treatment outcomes and identify novel targets for rehabilitation (e.g., memory for the content of treatment) that can be translated into clinical practice. Results from this study may also have wider clinical applicability, as they could lead to changes in other psychotherapeutic approaches for PTSD, as well as other disorders impacting cognition (e.g., traumatic brain injury). For example, “fine-tuned” psychotherapies could be developed to enhance memory for treatment content by providing focused memory support interventions, which could be broadly applied to help facilitate treatment effectiveness. One novel and promising pathway is to train therapists to infuse psychotherapy treatment-as-usual with a memory support intervention to enhance patient memory for the content of treatment (Harvey et al., 2014). This intervention involves therapist use of eight memory support strategies developed from cognitive psychology and education research on learning and memory, such as repeating information, writing down prescribed treatment recommendations, and providing cues to facilitate retrieval (see Harvey et al., 2014). Initial results from a clinical trial in depression indicate that memory support improves patient recall of treatment content and depression outcomes compared to cognitive-behavioral therapy alone (Harvey et al., 2016). Thus, if our hypotheses are confirmed, this intervention may be useful to adapt and test in future studies of CPT for PTSD.

## Ethics Statement

The studies involving human participants were reviewed and approved by the Corporal Michael J. Crescenz Veterans Affairs Institutional Review Board. The patients/participants provided their written or verbal informed consent to participate in this study.

## Author Contributions

JCS obtained funding and is responsible for the overall study concept and design. DC and JCS drafted the manuscript. AA, CD, KL, JG, and GH provided critical revision of the manuscript for important intellectual content. KL also provided technical and statistical support. All authors contributed to the article and approved the submitted version.

## Conflict of Interest

The authors declare that the research was conducted in the absence of any commercial or financial relationships that could be construed as a potential conflict of interest.
